# Supplementary material for: Identification of photoperiod-induced specific miRNAs in the adrenal glands of Sunite sheep (Ovis aries)
Source: Front Vet Sci. 2022 Jul 22;9:888207. doi: 10.3389/fvets.2022.888207 (PMC9354845; doi:10.3389/fvets.2022.888207)
Supplement: Supplementary file 1 [file Data_Sheet_1.ZIP › Supplementary file/miRDeep2 file.zip!/miRDeep2 file/Know_Structure/LP42A2/oar-let-7f.pdf]

miRBase precursor : oar-let-7f
Total read count : 632878
oar-let-7f read count : 632760
remaining reads : 118

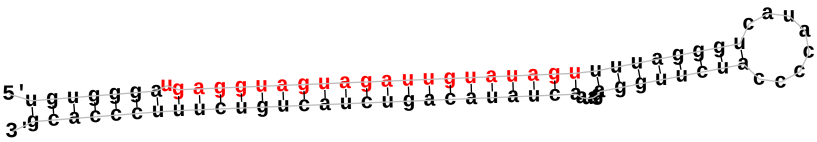

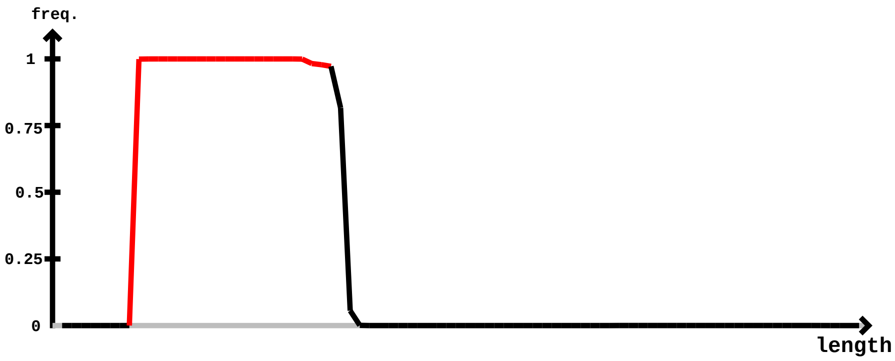


```
        oar-let-7f
5'- ugugggaugagguaguagauuguauaguuuuagggucauaccccaucuuggagauaacuauacagucuacugucuuucccacg   -3'   exp
    .((((((.((((((((((((((((((((((((((((........))))))))....)))))))))))))))))))))))))).    reads   mm   sample
    ......augagguaguagauuguauagu.......................................................    7       0    seq
    ......augagguaguagauuguauaguu......................................................    23      0    seq
    ......Gugagguaguagauuguauaguu......................................................    3       1    seq
    ......augagguaguagauuguauaguuu.....................................................    2       0    seq
    .......ugagguaguaAauugua...........................................................    1       1    seq
    .......ugagguaguagauugua...........................................................    354     0    seq
    .......ugaAguaguagauugua...........................................................    2       1    seq
    .......ugagguaguagauuguU...........................................................    5       1    seq
    .......ugagUuaguagauugua...........................................................    2       1    seq
    .......ugagguGguagauugua...........................................................    1       1    seq
    .......ugaggAaguagauugua...........................................................    1       1    seq
    .......ugagAuaguagauugua...........................................................    2       1    seq
    .......ugagguaguagauugAa...........................................................    1       1    seq
    .......ugaAguaguagauuguau..........................................................    89      1    seq
    .......ugagguaguagauuguUu..........................................................    7       1    seq
    .......ugagguaguagauuguau..........................................................    10500   0    seq
    .......ugagguaguUgauuguau..........................................................    2       1    seq
    .......ugagguaguagauugAau..........................................................    4       1    seq
    .......ugagguaguagauugAaua.........................................................    1       1    seq
    .......ugagguaguagauuguaua.........................................................    2723    0    seq
    .......ugagguaguagauugAauag........................................................    1       1    seq
    .......ugagguaguagauuguauag........................................................    3865    0    seq
    .......ugagguaguagauugAauagu.......................................................    15      1    seq
    .......ugagguaguagauuguauagu.......................................................    98444   0    seq
    .......ugagguaguagauuguauaguu......................................................    481039  0    seq
    .......ugagguaguagauuguauaguuG.....................................................    1526    1    seq
    .......ugagguaguagauuguauaguuu.....................................................    33250   0    seq
    .......ugagguaguagauuguauaguuuu....................................................    350     0    seq
    .......ugagguaguagauuguauaguuGu....................................................    97      1    seq
    .......ugagguaguagauuguauaguuuua...................................................    4       0    seq
    ........gagguaguagauuguau..........................................................    7       0    seq
    ........gagguaguagauuguaua.........................................................    1       0    seq
    ........gagguaguagauuguauag........................................................    2       0    seq
    ........gagguaguagauuguauagu.......................................................    63      0    seq
    ........gagguaguagauuguauaguu......................................................    287     0    seq
```

oar-let-7f

ugugggaugagguaguagauuguauaguuuuagggucauaccccaucuuggagauaacuauacagucuacugucuuucccacg			
........gagguaguagauuguauaguuu...	19	0	seq
.........agguaguagauuguauaguu..	6	0	seq
.........agguaguagauuguauaguuuu..	1	0	seq
.........agguaguagauuguauaguuGu..	45	1	seq
..........gguaguagauuguauaguu..	5	0	seq
...........guaguagauuguauaguu..	1	0	seq
............uaguagauuguauaguu..	2	0	seq
.............................uuagggucauaccccaucuugg................................	1	0	seq
.............................uuagggucauaccccaucuugga...............................	1	0	seq
.............................uuagggucauaccccaucuuggaga.............................	1	0	seq
....aacuauacagucuacugucuuuc.....	1	0	seq
....cuauacagucuacugucuuu......	10	0	seq
....cuauacagucuacugucuuuc.....	23	0	seq
....cuauacagucuacugucuuucc....	81	0	seq
